# First isolation of influenza D virus from cattle in Northeast China

**DOI:** 10.1128/spectrum.00374-24

**Published:** 2024-07-24

**Authors:** Hongbo Gao, Weiyang Sun, Pengyang Lu, Yuanguo Li, Juan Ren, Yeting Xia, Zhipeng Dong, Tiecheng Wang, Xianzhu Xia, Yuwei Gao

**Affiliations:** 1 Changchun Veterinary Research Institute, Chinese Academy of Agricultural Sciences, Changchun, Jilin, China; 2 Henan Provincial Engineering Center for Tumor Molecular Medicine, School of Basic Medical Sciences, Henan University, Kaifeng, China; 3 Ruminant Diseases Research Center, College of Life Sciences, Shandong Normal University, Jinan, China; Institute of Microbiology Chinese Academy of Sciences, Beijing, China

**Keywords:** influenza D virus, isolation, seroprevalence, Northeast China

## LETTER

Influenza D virus (IDV), first identified in 2011, has been detected in various animal species globally. Despite its broad host range, cattle are considered the natural reservoir of IDV (1). The first detection of IDV in China was made in Shandong Province, Eastern China (2). Subsequent reports of IDV in Southern China, including Guangdong (3, 4) and Nanjing provinces (5), followed. Notably, no infectious IDV was isolated in these cases. Herein, we present the first isolated IDV identified in bovine samples and conduct the IDV serosurveillance in Northeast China.

A total of 650 nasal swab samples were obtained from cattle herds in the cattle and sheep markets of Changchun, Jilin Province, from August to October 2023. We conducted real-time reverse transcription PCR (RT-PCR) on the collected samples following established protocols (6). And 12/650 samples tested positive for IDV. Subsequently, we employed RT-PCR methods to amplify the genome sequence of the positive samples (refer to Table S1). The genomic sequences of the 3/12 IDV-positive samples were obtained.

**Fig 1:**
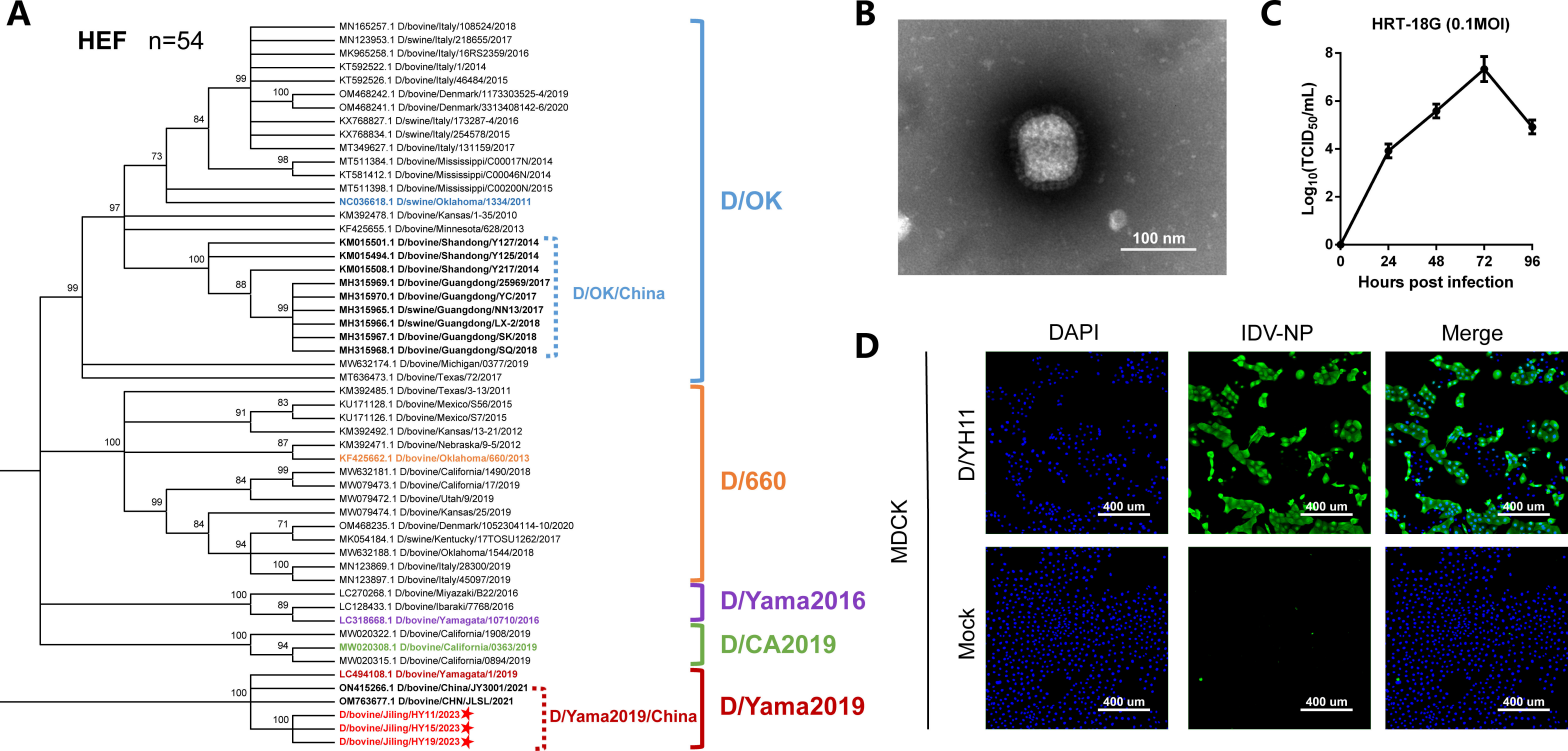
**(A**) Phylogenetic trees for the HEF segment of influenza D viruses (IDVs). Neighbor-joining phylogenetic tree of D/bovine/Jilin/HY11/2023 and other known IDV strains. Nucleotide sequences of HEF genetic segment were aligned and analyzed using MEGA XI, with 1,000 bootstrap replicates. Bootstrap scores of at least 70 were shown to the left of the major nodes. Each branch of five present lineages of IDV (D/OK-, D/660-, D/Yama2016-, D/CA2019-, and D/Yama2019-lineage) was noted by blue, orange, purple, green, and red, respectively. The strains described in this study were marked with a star. The strains identified in China were bolded. (**B**) Ultrastructural analysis of IDV isolates in cell cultures as observed by negative-staining electron micrography. Scale bar represents 100 nm. (**C**) Viral growth kinetics of D/HY11 strains in HRT18G cell cultures. We determined the virus titers in MDCK cells using the TCID_50_ values, shown as the means log_10_TCID_50_/mL ± SDs. (**D**) After 48 h of viral infection, cells were formalin-fixed and immunostained with the IDV-NP polyclonal antibody (green, customed from Sino Biological, Beijing, China) and 4′,6-diamidino-2-phenylindole (DAPI, Blue). Representative microscopy images of virus-infected and control cultures for MDCK cell lines are shown. Scale bar represents 400 µm.

We conducted a phylogenetic analysis of these IDV sequences utilizing maximum-likelihood analysis through MEGA-XI. Specifically, phylogenetic analysis of the hemagglutinin esterase fusion (HEF) segment was performed for representative IDVs (Fig. 1A). Supplementary to this, the phylogenetic trees for non-HEF segments of IDVs are presented in Fig. S1. The outcomes of the phylogenetic analysis revealed that the IDV sequences from this study formed a distinct cluster with previously reported sequences from China (3), aligning with the D/Yama 2019 lineage.

To isolate infectious IDV, human rectal tumor HRT-18G cells (CRL-11663; obtained from the American Type Culture Collection) were inoculated with the filtered (0.22 µm) supernatants from the virus transport medium of these positive samples. Subsequently, all samples were blindly passaged on HRT-18G cells (7) for 4–5 days at 37ºC. IDV RNA was detected using RT-PCR, and virus presence was confirmed in the cell culture supernatants of each passage by hemagglutination assays with 0.5% chicken RBC at room temperature (22–26ºC). Following four passages, virus-induced cytopathic effects became apparent and the virus that had been isolated was confirmed to be IDV (designated as D/bovine/Jilin/HY11/2023, abbreviated as HY11).

We also performed serological surveys using hemagglutination inhibition (HI) assay (8) with the HY11 strain as the antigen to ascertain the circulation of IDV among bovine populations in Northeast China. Of the 1,027 serum samples taken from cattle at routine slaughter in 2023, 678 samples were positive (HI titers equal to or higher than 40) for antibodies to IDV, resulting in an overall seroprevalence of 66.02% (Table 1). This was the first IDV serological survey with a prevalence rate that surpassed expectations conducted in the Chinese region.

**TABLE 1:** Distribution of IDV-positive antibody

HI^a^ titer	Number of samples	Percentage occupied (%)
<10	196	19.08
10	55	5.36
20	98	9.54
40	204	19.86
80	209	20.35
160	159	15.48
320	69	6.72
640	32	3.12
1280	5	0.49

^a^ Hemagglutination inhibition.

Furthermore, to gain a comprehensive understanding of the isolated IDV, we also performed assessments of its morphological features, growth kinetics, and immunofluorescence assay (Fig. 1B through D).

In summary, we successfully isolated an infectious IDV strain in a live cattle market, marking the inaugural IDV serosurveillance in Northeast China. Our findings suggest the widespread circulation of IDV in this region, emphasizing the need for further epidemiological investigations to evaluate the potential risks posed to both animal and human health.

## Data Availability

The genomic sequences of the three IDV-positive samples were designated D/bovine/Jilin/HY11/2023, D/bovine/Jilin/HY15/2023, and D/bovine/Jilin/HY19/2023. These sequences have been submitted to GenBank with accession no. OR880337-OR880345.

## SUPPLEMENTAL MATERIAL

Supplemental table and figure spectrum.00374-24-s0001.pdf Table S1 and Fig. S1.

ASM does not own the copyrights to Supplemental Material that may be linked to, or accessed through, an article. The authors have granted ASM a non-exclusive, world-wide license to publish the Supplemental Material files. Please contact the corresponding author directly for reuse.
