# Supplementary material for: First isolation of influenza D virus from cattle in Northeast China
Source: Microbiol Spectr. 2024 Jul 24;12(9):e00374-24. doi: 10.1128/spectrum.00374-24 (PMC11370236; doi:10.1128/spectrum.00374-24)
Supplement: Supplemental table and figure — Table S1 and Fig. S1. [file spectrum.00374-24-s0001.pdf]

Supplemental material

Supplemental Table 1. Primers for coding-complete amplification of seven segments of IDV*

**Primers**	**Sequences (5’-3’)**
IDV-HEF F	AGCATAAGCAGGAGATTTTCA
IDV-HEF R	AGCAGTAGCAAGGAGATTTTTTC
IDV-NS F	AGCATAAGCAGGGGTGTACA
IDV-NS R	CAGTAGCAAGGGGTTTTTTC
IDV-P42 F	GCATAAGCAGAGGATATTTTTG
IDV-P42 R	AGCAGTAGCAAGAGGATTTTTTC
IDV-NP F	GGAGATTATTAAGCAATATGGACTC
IDV-NP R	AAACCAACACCTTTAACACCC
IDV-P3 F	AGCATAAGCAGGAGATTTAGAAA
IDV-P3 R	AGCAGTAGCAAGGAGATTTTTAAC
IDV-PB1 F	GGCATAAGCAGAGGATT
IDV-PB1 R	AGCAGTAGCAAGAGGATTTTTC
IDV-PB2 F	AGCATAAGCAGAGGATGTCAC
IDV-PB2 R	AGCAGTAGCAAGAGGATTTTTTC

* Influenza D virus

Supplemental Figure 1

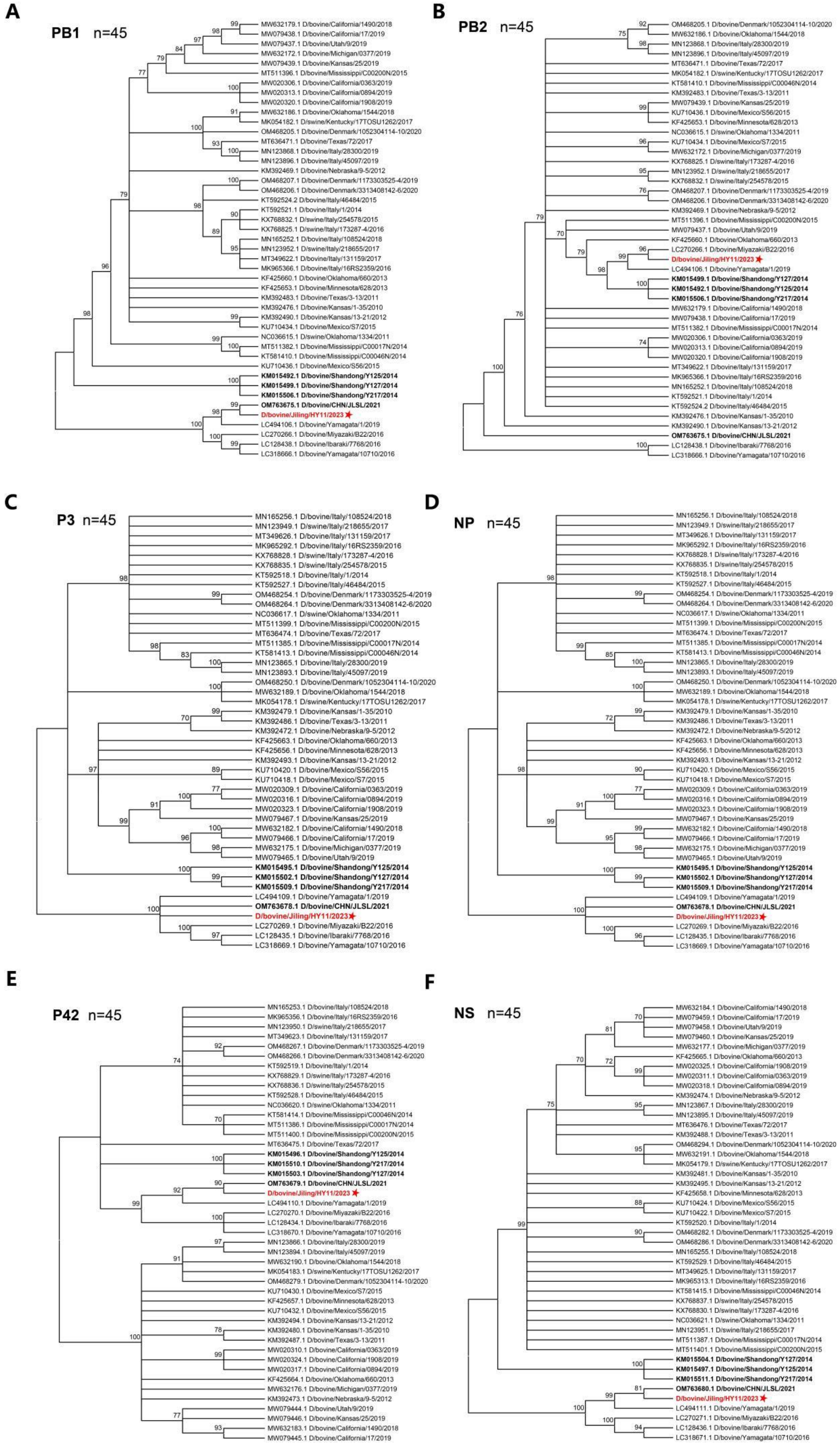
A
PB1 n=45
MW632179.1 D/bovine/California/1490/2018
MW079438.1 D/bovine/California/17/2019
MW079437.1 D/bovine/Utah/9/2019
MW632172.1 D/bovine/Michigan/0377/2019
MW079439.1 D/bovine/Kansas/25/2019
MT511396.1 D/bovine/Mississippi/C00200N/2015
MW020306.1 D/bovine/California/0363/2019
MW020313.1 D/bovine/California/0894/2019
MW020320.1 D/bovine/California/1908/2019
MW632186.1 D/bovine/Oklahoma/1544/2018
MK054182.1 D/swine/Kentucky/17TOSU1262/2017
OM468205.1 D/bovine/Denmark/1052304114-10/2020
MT636471.1 D/bovine/Texas/72/2017
MN123868.1 D/bovine/Italy/28300/2019
MN123896.1 D/bovine/Italy/45097/2019
KM392469.1 D/bovine/Nebraska/9-5/2012
OM468207.1 D/bovine/Denmark/1173303525-4/2019
OM468206.1 D/bovine/Denmark/3313408142-6/2020
KT592524.2 D/bovine/Italy/46484/2015
KT592521.1 D/bovine/Italy/1/2014
KX768832.1 D/swine/Italy/254578/2015
KX768825.1 D/swine/Italy/173287-4/2016
MN165252.1 D/bovine/Italy/108524/2018
MN123952.1 D/swine/Italy/218655/2017
MT349622.1 D/bovine/Italy/131159/2017
MK965366.1 D/bovine/Italy/16RS2359/2016
KF425660.1 D/bovine/Oklahoma/660/2013
KF425653.1 D/bovine/Minnesota/628/2013
KM392483.1 D/bovine/Texas/3-13/2011
KM392476.1 D/bovine/Kansas/1-35/2010
KM392490.1 D/bovine/Kansas/13-21/2012
KU710434.1 D/bovine/Mexico/S7/2015
NC036615.1 D/swine/Oklahoma/1334/2011
MT511382.1 D/bovine/Mississippi/C00017N/2014
KT581410.1 D/bovine/Mississippi/C00046N/2014
KU710436.1 D/bovine/Mexico/S56/2015
KM015492.1 D/bovine/Shandong/Y125/2014
KM015499.1 D/bovine/Shandong/Y127/2014
KM015506.1 D/bovine/Shandong/Y217/2014
OM763675.1 D/bovine/CHN/JLSL/2021
D/bovine/Jiling/HY11/2023
LC494106.1 D/bovine/Yamagata/1/2019
LC270266.1 D/bovine/Miyazaki/B22/2016
LC128438.1 D/bovine/Ibaraki/7768/2016
LC318666.1 D/bovine/Yamagata/10710/2016
B
PB2 n=45
OM468205.1 D/bovine/Denmark/1052304114-10/2020
MW632186.1 D/bovine/Oklahoma/1544/2018
MN123868.1 D/bovine/Italy/28300/2019
MN123896.1 D/bovine/Italy/45097/2019
MT636471.1 D/bovine/Texas/72/2017
MK054182.1 D/swine/Kentucky/17TOSU1262/2017
KT581410.1 D/bovine/Mississippi/C00046N/2014
KM392483.1 D/bovine/Texas/3-13/2011
MW079439.1 D/bovine/Kansas/25/2019
KU710436.1 D/bovine/Mexico/S56/2015
KF425653.1 D/bovine/Minnesota/628/2013
NC036615.1 D/swine/Oklahoma/1334/2011
KU710434.1 D/bovine/Mexico/S7/2015
MW632172.1 D/bovine/Michigan/0377/2019
KX768825.1 D/swine/Italy/173287-4/2016
MN123952.1 D/swine/Italy/218655/2017
KX768832.1 D/swine/Italy/254578/2015
OM468207.1 D/bovine/Denmark/1173303525-4/2019
OM468206.1 D/bovine/Denmark/3313408142-6/2020
KM392469.1 D/bovine/Nebraska/9-5/2012
MT511396.1 D/bovine/Mississippi/C00200N/2015
MW079437.1 D/bovine/Utah/9/2019
KF425660.1 D/bovine/Oklahoma/660/2013
LC270266.1 D/bovine/Miyazaki/B22/2016
D/bovine/Jiling/HY11/2023
LC494106.1 D/bovine/Yamagata/1/2019
KM015499.1 D/bovine/Shandong/Y127/2014
KM015492.1 D/bovine/Shandong/Y125/2014
KM015506.1 D/bovine/Shandong/Y217/2014
MW632179.1 D/bovine/California/1490/2018
MW079438.1 D/bovine/California/17/2019
MT511382.1 D/bovine/Mississippi/C00017N/2014
MW020306.1 D/bovine/California/0363/2019
MW020313.1 D/bovine/California/0894/2019
MW020320.1 D/bovine/California/1908/2019
MT349622.1 D/bovine/Italy/131159/2017
MK965366.1 D/bovine/Italy/16RS2359/2016
MN165252.1 D/bovine/Italy/108524/2018
KT592521.1 D/bovine/Italy/1/2014
KT592524.2 D/bovine/Italy/46484/2015
KM392476.1 D/bovine/Kansas/1-35/2010
KM392490.1 D/bovine/Kansas/13-21/2012
OM763675.1 D/bovine/CHN/JLSL/2021
LC128438.1 D/bovine/Ibaraki/7768/2016
LC318666.1 D/bovine/Yamagata/10710/2016
C
P3 n=45
MN165256.1 D/bovine/Italy/108524/2018
MN123949.1 D/swine/Italy/218655/2017
MT349626.1 D/bovine/Italy/131159/2017
MK965292.1 D/bovine/Italy/16RS2359/2016
KX768828.1 D/swine/Italy/173287-4/2016
KX768835.1 D/swine/Italy/254578/2015
KT592518.1 D/bovine/Italy/1/2014
KT592527.1 D/bovine/Italy/46484/2015
OM468254.1 D/bovine/Denmark/1173303525-4/2019
OM468264.1 D/bovine/Denmark/3313408142-6/2020
NC036617.1 D/swine/Oklahoma/1334/2011
MT511399.1 D/bovine/Mississippi/C00200N/2015
MT636474.1 D/bovine/Texas/72/2017
MT511385.1 D/bovine/Mississippi/C00017N/2014
KT581413.1 D/bovine/Mississippi/C00046N/2014
MN123865.1 D/bovine/Italy/28300/2019
MN123893.1 D/bovine/Italy/45097/2019
OM468250.1 D/bovine/Denmark/1052304114-10/2020
MW632189.1 D/bovine/Oklahoma/1544/2018
MK054178.1 D/swine/Kentucky/17TOSU1262/2017
KM392479.1 D/bovine/Kansas/1-35/2010
KM392486.1 D/bovine/Texas/3-13/2011
KM392472.1 D/bovine/Nebraska/9-5/2012
KF425663.1 D/bovine/Oklahoma/660/2013
KF425656.1 D/bovine/Minnesota/628/2013
KM392493.1 D/bovine/Kansas/13-21/2012
KU710420.1 D/bovine/Mexico/S56/2015
KU710418.1 D/bovine/Mexico/S7/2015
MW020309.1 D/bovine/California/0363/2019
MW020316.1 D/bovine/California/0894/2019
MW020323.1 D/bovine/California/1908/2019
MW079467.1 D/bovine/Kansas/25/2019
MW632182.1 D/bovine/California/1490/2018
MW079466.1 D/bovine/California/17/2019
MW632175.1 D/bovine/Michigan/0377/2019
MW079465.1 D/bovine/Utah/9/2019
KM015495.1 D/bovine/Shandong/Y125/2014
KM015502.1 D/bovine/Shandong/Y127/2014
KM015509.1 D/bovine/Shandong/Y217/2014
LC494109.1 D/bovine/Yamagata/1/2019
OM763678.1 D/bovine/CHN/JLSL/2021
D/bovine/Jiling/HY11/2023
LC270269.1 D/bovine/Miyazaki/B22/2016
LC128435.1 D/bovine/Ibaraki/7768/2016
LC318669.1 D/bovine/Yamagata/10710/2016
D
NP n=45
MN165256.1 D/bovine/Italy/108524/2018
MN123949.1 D/swine/Italy/218655/2017
MT349626.1 D/bovine/Italy/131159/2017
MK965292.1 D/bovine/Italy/16RS2359/2016
KX768828.1 D/swine/Italy/173287-4/2016
KX768835.1 D/swine/Italy/254578/2015
KT592518.1 D/bovine/Italy/1/2014
KT592527.1 D/bovine/Italy/46484/2015
OM468254.1 D/bovine/Denmark/1173303525-4/2019
OM468264.1 D/bovine/Denmark/3313408142-6/2020
NC036617.1 D/swine/Oklahoma/1334/2011
MT511399.1 D/bovine/Mississippi/C00200N/2015
MT636474.1 D/bovine/Texas/72/2017
MT511385.1 D/bovine/Mississippi/C00017N/2014
KT581413.1 D/bovine/Mississippi/C00046N/2014
MN123865.1 D/bovine/Italy/28300/2019
MN123893.1 D/bovine/Italy/45097/2019
OM468250.1 D/bovine/Denmark/1052304114-10/2020
MW632189.1 D/bovine/Oklahoma/1544/2018
MK054178.1 D/swine/Kentucky/17TOSU1262/2017
KM392479.1 D/bovine/Kansas/1-35/2010
KM392486.1 D/bovine/Texas/3-13/2011
KM392472.1 D/bovine/Nebraska/9-5/2012
KF425663.1 D/bovine/Oklahoma/660/2013
KF425656.1 D/bovine/Minnesota/628/2013
KM392493.1 D/bovine/Kansas/13-21/2012
KU710420.1 D/bovine/Mexico/S56/2015
KU710418.1 D/bovine/Mexico/S7/2015
MW020309.1 D/bovine/California/0363/2019
MW020316.1 D/bovine/California/0894/2019
MW020323.1 D/bovine/California/1908/2019
MW079467.1 D/bovine/Kansas/25/2019
MW632182.1 D/bovine/California/1490/2018
MW079466.1 D/bovine/California/17/2019
MW632175.1 D/bovine/Michigan/0377/2019
MW079465.1 D/bovine/Utah/9/2019
KM015495.1 D/bovine/Shandong/Y125/2014
KM015502.1 D/bovine/Shandong/Y127/2014
KM015509.1 D/bovine/Shandong/Y217/2014
LC494109.1 D/bovine/Yamagata/1/2019
OM763678.1 D/bovine/CHN/JLSL/2021
D/bovine/Jiling/HY11/2023
LC270269.1 D/bovine/Miyazaki/B22/2016
LC128435.1 D/bovine/Ibaraki/7768/2016
LC318669.1 D/bovine/Yamagata/10710/2016
E
P42 n=45
MN165253.1 D/bovine/Italy/108524/2018
MK965356.1 D/bovine/Italy/16RS2359/2016
MN123950.1 D/swine/Italy/218655/2017
MT349623.1 D/bovine/Italy/131159/2017
OM468267.1 D/bovine/Denmark/1173303525-4/2019
OM468266.1 D/bovine/Denmark/3313408142-6/2020
KT592519.1 D/bovine/Italy/1/2014
KX768829.1 D/swine/Italy/173287-4/2016
KX768836.1 D/swine/Italy/254578/2015
KT592528.1 D/bovine/Italy/46484/2015
NC036620.1 D/swine/Oklahoma/1334/2011
KT581414.1 D/bovine/Mississippi/C00046N/2014
MT511386.1 D/bovine/Mississippi/C00017N/2014
MT511400.1 D/bovine/Mississippi/C00200N/2015
MT636475.1 D/bovine/Texas/72/2017
KM015496.1 D/bovine/Shandong/Y125/2014
KM015510.1 D/bovine/Shandong/Y217/2014
KM015503.1 D/bovine/Shandong/Y127/2014
OM763679.1 D/bovine/CHN/JLSL/2021
D/bovine/Jiling/HY11/2023
LC494110.1 D/bovine/Yamagata/1/2019
LC270270.1 D/bovine/Miyazaki/B22/2016
LC128434.1 D/bovine/Ibaraki/7768/2016
LC318670.1 D/bovine/Yamagata/10710/2016
MN123866.1 D/bovine/Italy/28300/2019
MN123894.1 D/bovine/Italy/45097/2019
MW632190.1 D/bovine/Oklahoma/1544/2018
MK054183.1 D/swine/Kentucky/17TOSU1262/2017
OM468279.1 D/bovine/Denmark/1052304114-10/2020
KU710430.1 D/bovine/Mexico/S7/2015
KF425657.1 D/bovine/Minnesota/628/2013
KU710432.1 D/bovine/Mexico/S56/2015
KM392494.1 D/bovine/Kansas/13-21/2012
KM392480.1 D/bovine/Kansas/1-35/2010
KM392487.1 D/bovine/Texas/3-13/2011
MW020310.1 D/bovine/California/0363/2019
MW020324.1 D/bovine/California/1908/2019
MW020317.1 D/bovine/California/0894/2019
KF425664.1 D/bovine/Oklahoma/660/2013
MW632176.1 D/bovine/Michigan/0377/2019
KM392473.1 D/bovine/Nebraska/9-5/2012
MW079444.1 D/bovine/Utah/9/2019
MW079446.1 D/bovine/Kansas/25/2019
MW632183.1 D/bovine/California/1490/2018
MW079445.1 D/bovine/California/17/2019
F
NS n=45
MW632184.1 D/bovine/California/1490/2018
MW079459.1 D/bovine/California/17/2019
MW079458.1 D/bovine/Utah/9/2019
MW079460.1 D/bovine/Kansas/25/2019
MW632177.1 D/bovine/Michigan/0377/2019
KF425665.1 D/bovine/Oklahoma/660/2013
MW020325.1 D/bovine/California/1908/2019
MW020311.1 D/bovine/California/0363/2019
MW020318.1 D/bovine/California/0894/2019
KM392474.1 D/bovine/Nebraska/9-5/2012
MN123867.1 D/bovine/Italy/28300/2019
MN123895.1 D/bovine/Italy/45097/2019
MT636476.1 D/bovine/Texas/72/2017
KM392488.1 D/bovine/Texas/3-13/2011
OM468294.1 D/bovine/Denmark/1052304114-10/2020
MW632191.1 D/bovine/Oklahoma/1544/2018
MK054179.1 D/swine/Kentucky/17TOSU1262/2017
KM392481.1 D/bovine/Kansas/1-35/2010
KM392495.1 D/bovine/Kansas/13-21/2012
KF425658.1 D/bovine/Minnesota/628/2013
KU710424.1 D/bovine/Mexico/S56/2015
KU710422.1 D/bovine/Mexico/S7/2015
KT592520.1 D/bovine/Italy/1/2014
OM468282.1 D/bovine/Denmark/1173303525-4/2019
OM468286.1 D/bovine/Denmark/3313408142-6/2020
MN165255.1 D/bovine/Italy/108524/2018
KT592529.1 D/bovine/Italy/46484/2015
MT349625.1 D/bovine/Italy/131159/2017
MK965313.1 D/bovine/Italy/16RS2359/2016
KT581415.1 D/bovine/Mississippi/C00046N/2014
KX768837.1 D/swine/Italy/254578/2015
KX768830.1 D/swine/Italy/173287-4/2016
NC036621.1 D/swine/Oklahoma/1334/2011
MN123951.1 D/swine/Italy/218655/2017
MT511387.1 D/bovine/Mississippi/C00017N/2014
MT511401.1 D/bovine/Mississippi/C00200N/2015
KM015504.1 D/bovine/Shandong/Y127/2014
KM015497.1 D/bovine/Shandong/Y125/2014
KM015511.1 D/bovine/Shandong/Y217/2014
OM763680.1 D/bovine/CHN/JLSL/2021
D/bovine/Jiling/HY11/2023
LC494111.1 D/bovine/Yamagata/1/2019
LC270271.1 D/bovine/Miyazaki/B22/2016
LC128436.1 D/bovine/Ibaraki/7768/2016
LC318671.1 D/bovine/Yamagata/10710/2016

Supplemental Figure Legend

Supplemental FIG 1. Phylogenetic trees for non-HEF segments of IDVs. In MEGA-XI, maximum-likelihood analysis in combination with 1,000 bootstrap replicates was used to generate trees based on the nucleotide sequences of the PB1, PB2, P3, NP, P42 and NS segments (A-F). The strain isolated in this study (D/bovine/Jilin/HY11/2023) was marked with a star, and the strains identified in China were bolded.
